# Performance in Behavioral Testing in an Animal Model of Post-Surgical Hypoparathyroidism

**DOI:** 10.3390/jpm14020215

**Published:** 2024-02-17

**Authors:** Cristina Dettori, Francesca Ronca, Giulia Di Buono, Alessandro Saba, Francesca Di Lupo, Beatrice Polini, Caterina Ricardi, Sabina Frascarelli, Filomena Cetani, Claudio Marcocci, Riccardo Zucchi, Grazia Chiellini, Marco Scalese, Federica Saponaro

**Affiliations:** 1 Department of Surgical, Medical and Molecular Pathology and Critical Care, 56126 Pisa, Italy; c.dettori@studenti.unipi.it (C.D.); alessandro.saba@unipi.it (A.S.); f.dilupo1@studenti.unipi.it (F.D.L.); riccardo.zucchi@unipi.it (R.Z.); grazia.chiellini@unipi.it (G.C.); 2 Endocrine Unit, University Hospital of Pisa, 56124 Pisa, Italy; cetani@endoc.med.unipi.it (F.C.);; 3 Institute of Clinical Physiology, National Council of Research, 56126 Pisa, Italy

**Keywords:** PTH, hypoparathyroidism, post-surgical hypoparathyroidism

## Abstract

Background: Hypoparathyroidism (HypoPT) is characterized by hypocalcemia and undetectable/inappropriately low PTH. Post-surgical HypoPT (PS-HypoPT) is the most common cause. Patients with PS-HypoPT present neuropsychological symptoms, probably due to the PTH deprivation in the central nervous system (CNS). However, these mechanisms are still not elucidated. The aim of this study was to evaluate the effects of PTH deprivation on CNS in an animal model of PS-HypoPT via a cognitive/behavioral assessment approach. Methods: A surgical rat model of PS-HypoPT was obtained and treated with calcium to maintain normocalcemia. Twenty PS-HypoPT rats and twenty sham-operated controls (Crl) underwent behavioral testing in a Morris Water Maze (MWM), Open Field (OF), and Elevated Plus Maze (EPM). Results: In the MWM, PTx rats showed a higher Escape Latency Time compared to Crl rats (*p* < 0.05); we observed a statistically significant improvement in the performance (day 1 to 8 *p* < 0.001), which was less pronounced in PTx group. In the OF test, the time and distance spent in the zone of interest were significantly lower in the PTx group compared with the Crl (*p* < 0.01 and *p* < 0.01). In the EPM experiment, the time spent in the close arm was significantly higher in the PTx group compared with the Crl (*p* < 0.01). Conclusions: This animal model of PS-HypoPT shows an impairment in spatial memory, which improved after training, and a marked anxiety-like behavior, resembling the condition of patients with PS-HypoPT. Further studies are needed to elucidate mechanisms.

## 1. Introduction

Hypoparathyroidism (HypoPT) is defined as a relatively uncommon endocrine disorder characterized by the presence of low levels of calcium in the bloodstream, a condition known as hypocalcaemia, alongside undetectable or inappropriately low levels of serum parathyroid hormone (PTH) [1]. The predominant cause of HypoPT is frequently post-surgical in nature, stemming from unintended parathyroid removal or injury during neck surgery. PTH, a fundamental player in the intricate calcium homeostasis, exerts its influence in various physiological aspects, including the kidney reabsorption of calcium and phosphate excretion, regulatory functions in skeletal bone turnover, and an indirect impact on gastrointestinal calcium absorption mediated by vitamin D [2].

The absence of PTH in individuals with HypoPT leads to a significant change in calcium homeostasis, exposing them to the risks of hypocalcaemia, hypercalciuria, hyperphosphatemia, and the abnormal mineralization of the skeletal structure [3,4]. This cascade of biochemical disruptions culminates in persistent clinical manifestations that affect multiple organs, leading to conditions like nephrolithiasis and to a gradual decline in kidney function, to a compromised bone turnover and a diminished bone quality, to a heightened susceptibility to cardiovascular diseases, neuromuscular impairment, and neuropsychological symptoms [5,6]. Conventional therapeutic interventions, typically relying on calcium supplements and activated vitamin D, often fall short of achieving a complete restoration of calcium balance [7]. A notable stride in medical progress has been made with the regulatory approval of recombinant human PTH (1–84) [rhPTH(1–84)] as an alternative treatment for patients who find conventional therapy inadequate [2,8,9].

From a neuropsychological standpoint, patients with PS-HypoPT present with a spectrum of cognitive and affective symptoms, including, but not limited to, brain fog, impaired focus, memory loss, depression, anxiety, and pervasive fatigue [10,11,12,13]. For this reason, several studies consistently corroborate a reduction in quality of life (QoL) among HypoPT patients when compared to both the general population and appropriately matched controls. While widely recognized self-administered questionnaires like the 36-Item Short Form Health Survey (SF-36), WHO-5 Well-being Index Survey (WHO-5), and Hospital Anxiety and Depression Scale have been extensively utilized, it is crucial to acknowledge their lack of specificity for HypoPT. The landscape has evolved with the introduction of novel, disease-specific questionnaires designed to specifically address HypoPT but still are not internationally validated [12,14,15,16,17].

However, the mechanisms by which the disease affects the Central Nervous System (CNS) have not yet been elucidated, and it is not clear whether they are related to an inadequate therapy or dependent on PTH deprivation itself. Indeed, the effects of PTH and PTH-related peptides on the CNS are very intriguing but poorly investigated and understood, in contrast to the well-known effects on classical target organs such as bones and kidneys.

The PTH family includes PTH, PTHrP, and tuberoinfundibular peptide of 39 (TIP39 or PTH2). These ligands can bind with different affinities to the parathyroid receptor type 1 (PTH1R) and type 2 (PTH2R). The PTH/PTHrP/PTH1R system is expressed in many areas of the brain (hippocampus, amygdala, hypothalamus, caudate nucleus, corpus callosum, subthalamic nucleus, thalamus, substantia nigra, and cerebellum) and the literature data suggest a protective action against neuroinflammation and neurodegeneration with positive effects on memory and hyperalgesia [18,19,20]. TIP39/PTH2 is a small peptide belonging to the PTH-related family with high affinity to PTH2R, specifically in the CNS. The TIP39/PTH2R system has been proposed to mediate many regulatory and functional roles in brain and to modulate auditory, nociceptive, and sexual maturation functions [21,22,23,24]. The protective effects of PTH and PTH-related peptides on the CNS have been demonstrated in in vitro and in vivo models [25], but to the best of our knowledge, they have not been studied in the context of hypoparathyroidism.

With these premises, reproducible animal models of hypoparathyroidism are necessary to dissect the specific effects of PTH deprivation, particularly in the view of treatments such as human recombinant PTH (rhPTH) administration, which has become available but still used only with few indications [8].

The aim of this study is to evaluate an animal model of surgical hypoparathyroidism from a behavioral point of view. A battery of behavioral tests was performed to analyze different cognitive features using a statistically relevant cohort.

## 2. Materials and Methods

### 2.1. Animals

Forty male Sprague–Dawley rats (Charles River Laboratories, France) were divided into two groups; namely, *n* = 20 underwent parathyroidectomy surgery, and *n* = 20 were sham operated. Parathyroidectomy and sham operations were performed at Charles Rivers Srl at 5 weeks of age and checked for PTH drop-out after surgery to undetectable levels. Upon arrival at the housing facility (6 weeks of age), the animals were acclimatized to the facility environment for 2 weeks before starting the behavioral tests. Following the surgical procedure, the parathyroidectomized rats were administered 1% calcium gluconate (Calciforte 500 mg, Grimberg Laboratoires) in 500 mg of drinking water refreshed every 3 days, to maintain normocalcemia (PTx rat). Drinking intake was monitored in PTx and control animals (Crl rat).

Rats’ development was monitored by weighing the animals once a week, and each rat was habituated to experimental handling through a 2 min session every 2 days.

All rats were kept in the same colony room at constant temperature (22 °C) and humidity (50%) on a reversed 12 h light/dark cycle, which was used to perform the experiments during the dark cycle when the rats have the highest activity.

All the experiments were conducted in compliance with the European Community Council Directive of 24 November 1986 (86/609/EEC) and in compliance with L.D. 4 March 2014 No. 26 for minimizing animal suffering and to use only the number of animals necessary to produce reliable results. The Italian Ministry of Health approved the use of animals in this protocol (approval number: 260/2019PR; A039F.4).

### 2.2. Biochemical Testing

Serum calcium was measured at 8 weeks of age, before behavioral testing, to confirm normocalcemia in PTx animals and ensure comparable levels of calcemia between PTx and Crl rats. Tail blood was collected, and serum was obtained after centrifugation at 1000× *g* for 15 min at 4 °C. Serum was stored at −20 °C and analyzed for total calcium levels using a colorimetric Calcium Assay Kit (Cayman Chemical, Ann Arbor, MI, USA).

### 2.3. Behavioral Testing

The day of the tests, the rats were transferred to the testing room at least 60 min before the beginning of the experiments. Rat behavior was monitored and recorded through an overhead camera, positioned above the Morris Water Maze (MWM), Open Field (OF), and Elevated Plus Maze (EPM) apparatus. The videos generated during the EPM were scored manually in a blind fashion by three independent observers. The videos generated during MWM and OF were analyzed by a dedicated software (SMART.3.0, Panlab, Cornella, Barcelona).

#### 2.3.1. Morris Water Maze Test (MWM)

The Morris Water Maze was used to test spatial memory and long-term memory by measuring the time of escape latency (time required to reach the platform), thigmotaxis duration, distance moved, and velocity during the time spent in the MWM water tank. The Morris Water Maze consisted of a round pool (diameter: 140 cm; height: 60 cm) filled with opaque water kept at 25 °C. The pool was virtually divided into four quadrants of equal area and a circular escape platform (10 cm diameter and 30–35 cm height) was placed in the center of one quadrant and its position was maintained throughout the testing. Four different visual clues were added externally around the circumference of the tank to help the animal to remember the position of the platform in relation to surrounding cues. The rat was given four acquisition trials per day for seven consecutive days. During the first two days of the acquisition training, the platform was located 1 cm above the water. On day one, the rat was first placed on the platform for 15 s before being released into the water at water-level facing the tank wall. The rat was given a maximum of 60 s to find the platform and was allowed to stay on it for 15 s during the inter-trial interval to allow to memorize its position in space. Four different starting positions were used for each animal. Rats that failed to locate the platform were placed onto the platform by the experimenter. During days three to seven of the acquisition training, the platform was hidden 1 cm below water.

After acquisition training, on the eighth day, each animal was given a probe trial, during which the platform was removed, and each animal was allowed 60 s to search the pool for the escape platform. The time and the number of times a subject took to intersect the area where the platform was previously located was measured and compared with the time spent in the other quadrants (platform crossing) and with the latencies obtained in the acquiring trials.

The Escape Latency Time, represented by the time spent reaching the platform (or, on the last probe day, the platform quadrant) was used as the main parameter [26].

#### 2.3.2. Open Field (OF)

The standard open field test is commonly used to assess locomotor, exploratory, anxiety-like behaviors, behavioral responses to novelty in laboratory animals, and depressive behaviors [27,28,29]. The open field test examines the natural opposite tendencies to explore a novel environment and the tendency to avoid a brightly lit area at the center of the field open area. When anxious, rodents tend to avoid discovery and stay put or move along the walls (thigmotaxis).

Each rat was placed at the center of the square open arena (90 cm × 90 cm) for 10 min and its behavior was videorecorded. The following parameters were scored by dedicated software: time and distance spent in outer zones versus inner zones (area of interest) [30].

#### 2.3.3. Elevated Plus Maze (EPM)

The EPM apparatus consisted of 4 arms (25 × 5 cm) forming a plus sign, elevated 50 cm above the floor, with two open and two closed walls. Each rat was allowed to freely explore the maze for 5 min. The time spent in open arms and in closed arms were manually scored in a blind fashion by three independent observers. The EPM measures the conflict between two opposite natural instincts of exploring or avoiding open, unprotected spaces. The higher the “anxiety” levels, the lower the proportion of explorations of open spaces (open arms) versus dark spaces (closed arms). Increased time spent in open arms indicates a lower degree of “anxiety” in the animals. At the end of each individual trial, the maze was cleaned with a 15% ethanol solution to remove olfactory cues.

### 2.4. Statistical Analysis

Categorical variables are stated as number (*n*) and percentage (%). Continuous variables are expressed as mean and standard deviation. Fisher exact or Chi-square tests were used for the comparisons of categorical variables. Mann–Whitney and Kruskal–Wallis tests were used to compare continuous variables. Multivariate linear regression was applied to assess whether performance could be dependent on treatment and time. A *p*-value < 0.05 was considered statistically significant.

## 3. Results

Both PTx (*n* = 20) and Crl rats (*n* = 20) showed normal growth, and there was not a statistically significant difference in body weight among the two groups at the beginning of behavioral testing (PTx 353.2 ± 19.1 g vs. Crl 400.6 ± 13.3 g, *p* = 0.7). Serum calcium levels were measured at 8 weeks of age; no statistically significant difference between PTx rats (supplemented with 1% calcium) and Crl rats was found (serum calcium: PTx 10.4 ± 0.5 mg/dL vs. 10.3 ± 0.2 mg/dL, *p* > 0.05).

### 3.1. Morris Water Maze Performance

**Figure 1:**
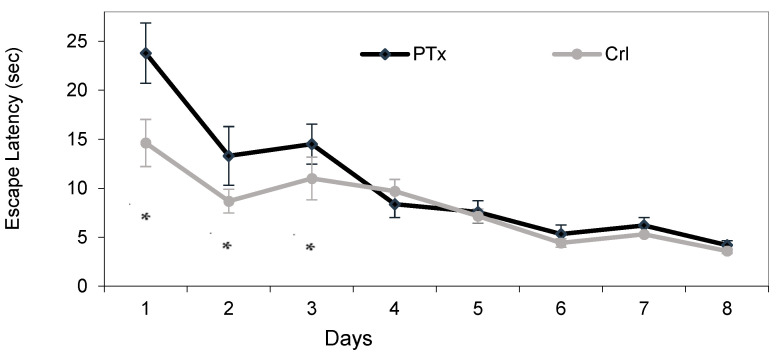
Morris Water Maze (MWM) test. Escape latency time (seconds) expressed as mean and standard deviation (SD) is shown for each day (day 1–day 8) of the MWM test in animals with post-surgical hypoparathyroidism (PTx, *n* = 20) and sham-operated controls (Crl, *n* = 20). * *p* < 0.05.

PTx rats showed a worse performance in the MWM test during the training period (day 1–3) compared to Crl rats, namely a higher escape latency time (sec., day 1 *p* = 0.02; day 2 *p* = 0.04, day 3 *p* = 0.01) (Figure 1).

In both groups, we observed a statistically significant improvement in the performance between day 1 and day 8, namely a significant reduction in escape latency (PTx group: 23.8 ± 3.1 vs. 4.2 ± 2.5 s; Crl group 14.6 ± 2.4 vs. 3.6 ± 1.4 s, *p* < 0.001).

In the MWM test, the PTx group reached the same performance as the Crl after the first three days of training and no statistically significant difference in escape latency time was observed on the last day of the test (probe test or Day 8: PTx 4.2 + 2.5 s vs. Crl 3.6 + 1.4 s, *p* = 0.1).

In the multivariate analysis including time and treatment (PTx or Crl), we observed that there was a significantly lower reduction in escape latency (sec) from the first to last day in the PTx animals compared to the Crl animals (Table 1), indicating that the PTx animals showed a lower improvement during the test.

These data suggest that although the animals with PS hypoparathyroidism showed an impaired spatial memory at the beginning of the test, they can recover and improve after training, although less than Crl rats.

### 3.2. Open Field and Elevated Plus Maze Performance

**Figure 2:**
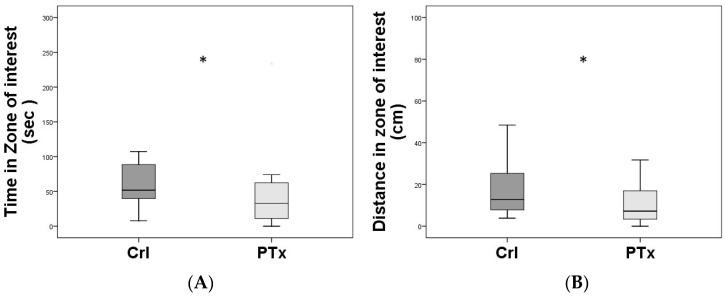
Open Field (OF) test. (**A**): Time spent in zone of interest in the OF, namely the center of the arena, expressed as means and SD, is significantly lower in PTx animal group (post-surgical HypoPT, *n* = 20), compared to Crl (control animal group sham operated, *n* = 20) (* *p* < 0.01). (**B**): The distance travelled in the zone of interest is significantly lower in the Ptx group compared to Crl (* *p* < 0.01).

In the Open Field test (OF), the time and the distance spent in the zone of interest (center of arena) were significantly lower in the PTx group (respectively *p* < 0.01 and *p* < 0.01), compared with the Crl group (Figure 2), suggesting that PTx animals show higher anxiety-like behavior.

**Figure 3:**
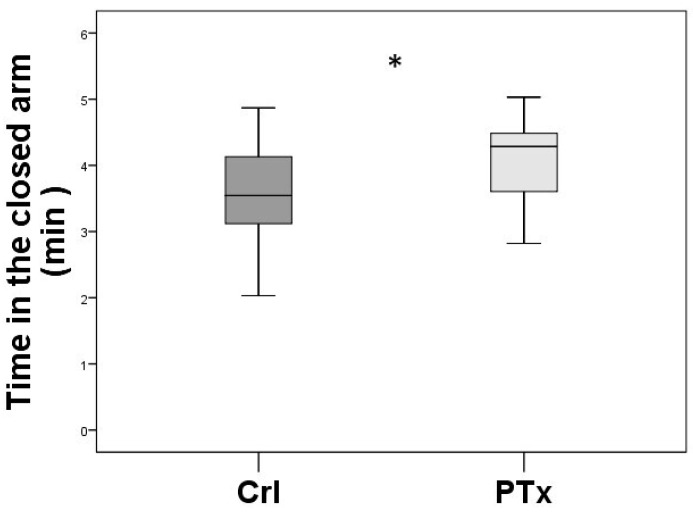
Elevated Plus Maze (EPM) test. Time spent (minutes) in the closed arm of EPM, expressed as means and SD, is significantly higher in PTx animal group (post-surgical HypoPT, *n* = 20), compared to Crl (control animal group sham operated, *n* = 20) (* *p* < 0.01).

In the Elevated Plus Maze (EPM) test, the main parameter, namely the time spent in the closed arm (min), was significantly higher in the PTx group (*p* < 0.01), compared with the Crl group (Figure 3), suggesting a higher anxiety-like behavior in the PTx group.

## 4. Discussion

Mineral metabolism is crucial in human health, and a complex hormonal system is associated to its maintenance, with a pivotal role played by PTH and PTH-related peptides. Despite the well-known role of PTH as the main modulator of calcium homeostasis, this vision turned out to be simplistic, since many other effects of this hormone have been hypothesized and demonstrated on different organ and tissue targets. The effect of PTH and PTH-related peptides on the CNS is sustained by preclinical and clinical evidence [25]. However, what happens to cognitive performance and brain function in those pathological conditions of PTH deprivation, such as PS-HypoPT, still need to be elucidated.

In the present study, we evaluated an animal model of PS-HypoPT from a cognitive point of view, mimicking the condition of patients with the disease. We demonstrated by different behavioral tests that animals with PS-HypoPT showed an impairment of spatial memory that improved after training and a marked anxiety-like behavior. To the best of our knowledge, this is the first study to evaluate behavioral performance in a model of chronic post-surgical hypoparathyroidism.

Due to the technical difficulties and the rarity of the disease, few animal models of hypoparathyroidism exist [31]. Some genetic models have been obtained in order to study either the idiopathic or the syndromic or the genetic variants of HypoPT; an example is the knockout model of mice deficient in either the genes *Crkol (Crkol−/−)* or *Tbx1 (Tbx1−/−),* which showed the same severe abnormalities of the DiGeorge syndrome, including hypoplasia of the parathyroid gland [32,33,34,35]. Another model of mild HypoPT with modest hypocalcemia is the mouse model presenting a deficiency in the gene *Gcm2*, which is part of the *GCMB* family of master regulators of parathyroid development, both in humans and in mice [36].

However, the physiopathology of these conditions is very different from the acquired chronic, post-surgical HypoPT. Indeed, the surgical model is the ideal one to summarize the pathophysiology of PS-HypoPT. In this study, we chose to use the rat as the animal model, since it is technically easier to remove only the parathyroid gland without damaging the thyroid gland in rats compared to mice, due to the animal size. Through adequate calcium supplementation, this PS-HypoPT rat model showed a normal growth rate and normal calcemia, allowing us to dissect PTH-specific deprivation effects on behavior.

Another surgical model has been described by using a 5-animolevulinic (5-ALA) fluorescent dye to identify and remove the entire parathyroid tissue [37]. This model proposed by Soo Yeon et al. [37] showed the same features as our surgical model, with similar calcium levels, despite the different calcium supplement. However, PTx animals reached a smaller weight compared to the controls, while in our study, the PTx group and Crl group did not statistically differ for weight and showed normal growth. In Soo Yeon et al.’s, study, PTx animals showed similar kidney and bone complications as are expected in HypoPT, but they were not evaluated from a CNS or behavioral point of view [37].

In our model, we employed a battery of cognitive behavioral tests. To assess spatial memory and learning, we used the Morris Water Maze (MWM). Since its development 40 years ago, the MWM has been improved and widely used to assess cognitive features. It is considered a valuable tool and the most reliable test to explore learning and the specific hippocampus-dependent spatial memory, void of the motivational differences across diverse experimental manipulations and applicable to different animal species [26]. We showed a decrease in spatial memory of PTx animals compared to Crl animals, even though the training was effective in improving performance. These data are well in accordance with the results of our previous study in patients with PS-HypoPT, where we evidenced reduced scores in spatial and verbal memory, evaluated with a neuropsychologist-trained approach [10]. Differently from idiopathic HypoPT, in which a severe cognitive impairment has been shown, in PS-HypoPT, the cognitive involvement is more nuanced, probably because of the longer and more pronounced PTH deprivation in the idiopathic variant [11]. However, even in PS-HypoPT, difficulty in concentration, in learning, in memory, and the so called “brain fog” are frequently reported symptoms in patients, accounting for reduced quality of life [14,38].

Patients with PS-HypoPT have also been shown to present an increase in the scores for anxiety, phobic anxiety, and their physical equivalents in some questionnaires, even though this remains controversial since other questionnaires did not show differences compared to healthy people [2]. It is noteworthy that the evaluation of anxiety in humans has the limitation of self-administered questionnaires which are generally not specific and objective, underlying the utility of animal models to study this aspect.

Open field and elevated plus maze tests explore anxiety-like behavior and show a high reliability, particularly when performed together [30,39]. A recent study showed how EPM is less influenced by environmental influences and age, whereas OF is less stressful for rodents [40]. In summary, anxiety-related behavior in animal models is a multidimensional structure and it comprises both simple components and functional interactions, as clearly shown by Carola et al. [41]. Indeed, this evidence stresses the importance of consequentially performing the two tests, which catch different and complementary aspects of anxiety-like behavior. In our study, both tests converged into a higher anxious behavior of PTx animals compared to the Crl animals. In the literature, anxiety-like behavior evaluated by EPM and OF has been related to the involvement of the basolateral amygdala (BLA) and the medial prefrontal cortex (mPFC), which are crucial neural hubs for the modulation of anxiety and emotionally driven performance [42]. In a recent study, Felix-Ortiz et al., by using an optogenetic approach to assess behavioral performance in OF and EPM to activate or inhibit the input of BLA toward mPFC, showed a causal relationship between BLA modulation and performance behavior in OF and EPM [43].

Although further studies on the brains of animal models of PS-HypoPT are much needed, we can hypothesize that memory impairment and anxiety behavior in PS-HypoPT could be related to PTH deprivation effects on the hippocampus and amygdala, even if mechanisms are yet to be elucidated.

PTH distribution in the brain has been well documented and it has been reported that PTHR1 and PTHR2 receptors are widely expressed in those areas of the brain and that PTH crosses the blood–brain barrier [25]. However, the local effects of PTH in the brain have not been well-explored. In the 1980s, studies demonstrated the direct action of PTH on memory and learning in rats; more recent research investigated PTH’s protective effect on memory in a mouse model of Alzheimer’s Disease (AD). The study found that treating mice with a PTH analogue led to improved memory, reduced neurodegeneration markers, and decreased neuroinflammation, suggesting a potential impact on brain astrocytes [44]. Additionally, PTH and its related peptide PTHrP have been implicated in modulating the cerebrovascular system. PTH can enhance neuroangiogenesis and neuroblast migration in ischemic cortical tissue, with PTHrP acting as a modulator of cerebral vasculature [45]. These findings indicate potential neuroprotective roles for PTH and PTHrP. Another proposed mechanism for PTH local action in the brain involves the paracrine modulation of catecholamine metabolism. Studies showed that the intracerebral administration of PTH influenced glutamic acid decarboxylase activity and increased the DOPAC/dopamine ratio in specific brain regions. Furthermore, PTH has been associated with hyperalgesia modulation in the brain, as suggested in earlier research and confirmed in recent studies using a PTH analogue [25]. These findings suggest that PTH may act on central neurons, offering a potential explanation for the relief of back pain observed in osteoporosis patients treated with PTH analogues [9]. Overall, these diverse actions of PTH in the brain warrant further investigation to better understand its potential neuroprotective effects [25].

Effects on the hippocampus and amygdala could be also mediated by some PTH-related peptides, such as TIP39, which are widely expressed in many areas of the CNS and still not completely elucidated in their physiological role [46,47,48].

In this study, which represents an interesting premise for further studies, we evaluated the potential effects of PTH deprivation on behavior. The strength of this study resides in the novelty of the use of a reliable animal model of PS-HypoPT in which, for the first time, to the best of our knowledge, a complete behavioral battery of tests has been performed. This study has also some limitations: we could measure and take under control only serum calcemia, confirming that there were no statistically significant differences between PTx and Crl groups, which could be responsible for behavioral changes. However, we could not measure vitamin D and phosphate, which in turn can display an effect on electrolyte balance and subsequently on brain function.

## 5. Conclusions

In conclusion, clinical evidence pushes the research toward a more critical and in-depth analysis of the extraskeletal effects of PTH, accounting for possible beneficial effects of novel substitutive therapy. This study suggested that PTH deprivation might have a role in brain function and in the response to memory inputs and stress. Our findings open the way to further molecular and histochemical studies to assess the physiopathology of PTH deprivation in the brain.

## Figures and Tables

**Table 1:** Multivariate analysis for the variable escape latency (s) including time (days of the MWM test) and treatment (PTx vs. Crl).

	β	*p*
Treatment (Crl vs. PTx)	−7.2	0.002
Time (Day 1 vs. Day 8)	−2.5	0.000
Treatment/Time	1.16	0.04

## Data Availability

The data presented in this study are available on request from the corresponding author.
